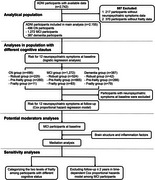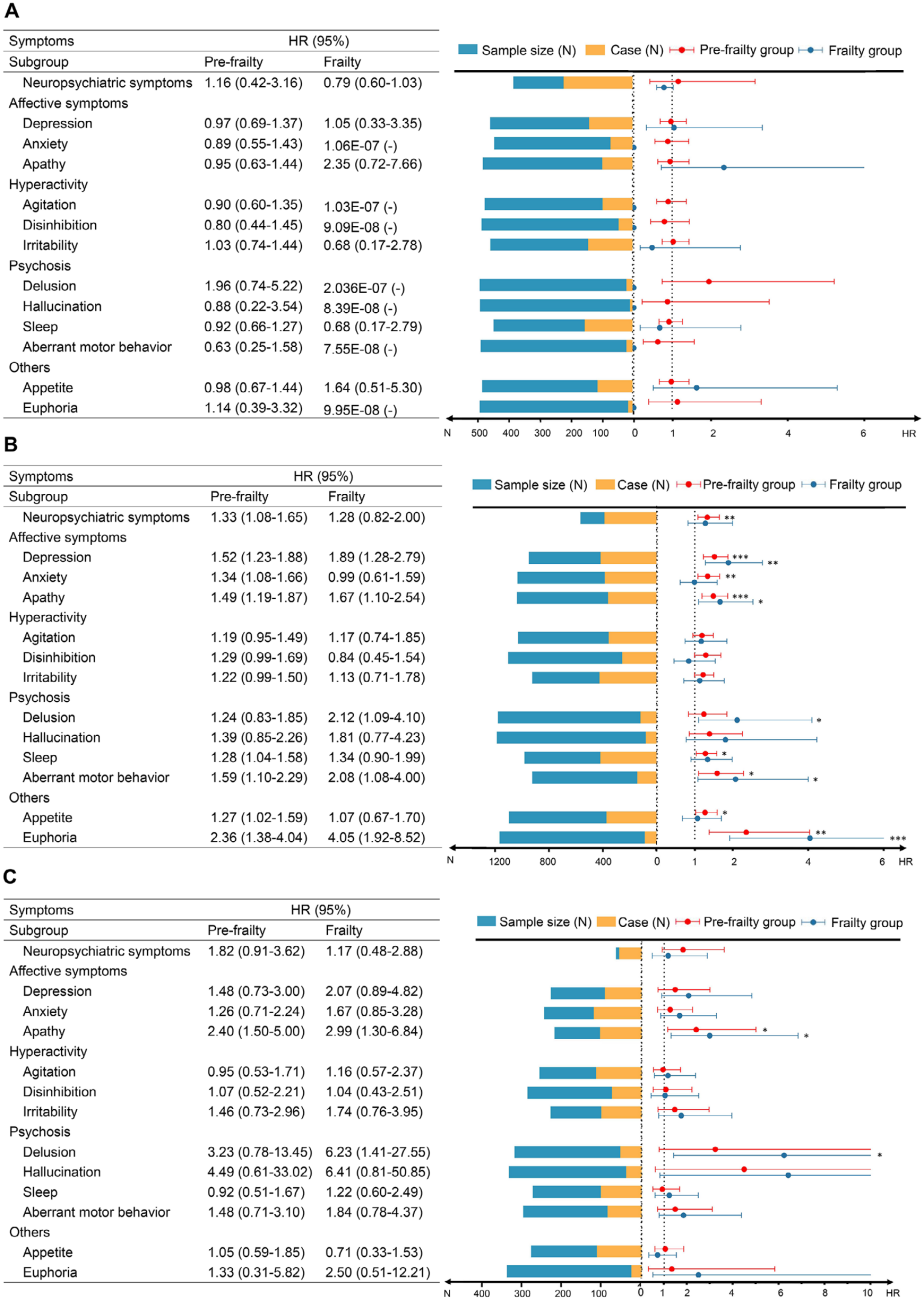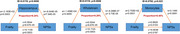# Associations of frailty with neuropsychiatric symptoms of Alzheimer's Disease: a longitudinal study

**DOI:** 10.1002/alz70860_100895

**Published:** 2025-12-23

**Authors:** Hao‐chen Chi, Lan Tan, Jin‐tai Yu

**Affiliations:** ^1^ Qingdao Municipal Hospital, Qingdao, Shandong, China; ^2^ Qingdao Municipal hospital, Qingdao university, Qingdao, Shandong, China; ^3^ Huashan Hospital, Fudan University, Shanghai, Shanghai, China

## Abstract

**Background:**

Frailty is a vulnerability state increasing the risk of many adverse health outcomes, but little is known about the effects of frailty on neuropsychiatric health.

**Method:**

We included 2,155 individuals assessed using modified frailty index‐11 (mFI‐11), Neuropsychiatric Inventory (NPI) and Neuropsychiatric Inventory Questionnaire (NPI‐Q) in the Alzheimer's Disease Neuroimaging Initiative (ADNI). The relationships between frailty andneuropsychiatric symptoms (NPSs) were explored with logistic regression models and Cox proportional hazard regression models. Causal mediation analyses were conducted to explore the mediation factors between frailty and NPSs.

**Result:**

Among mild cognitive impairment (MCI) participants, frailty was cross‐sectionally associated with an increased risk of apathy, and longitudinally associated with increased risk of depression and apathy. Among AD participants, frailty was cross‐sectionally associated with increased risk of depression and anxiety, and longitudinally associated with an increased risk of apathy. Among participants with cognitive progression, frailty was associated with increased risk of depression and apathy. In MCI participants, the influence of frailty on NPSs was partially mediated by hippocampus volume, whole brain volume, and monocytes, with mediating proportions ranging from 8.40% to 9.29%.

**Conclusion:**

Frailty was associated with NPSs such as depression, anxiety, and apathy among MCI, AD, and cognitive progression participants. Atrophy of the hippocampus and whole brain, as well as peripheral immunity may be involved in the potential mechanisms underlying the above associations.